# Supplementary figures and images for: Resistance profiling of Aspergillus fumigatus to olorofim indicates absence of intrinsic resistance and unveils the molecular mechanisms of acquired olorofim resistance
Source: Emerg Microbes Infect. 2022 Mar 1;11(1):703–14. doi: 10.1080/22221751.2022.2034485 (PMC8890541; doi:10.1080/22221751.2022.2034485)

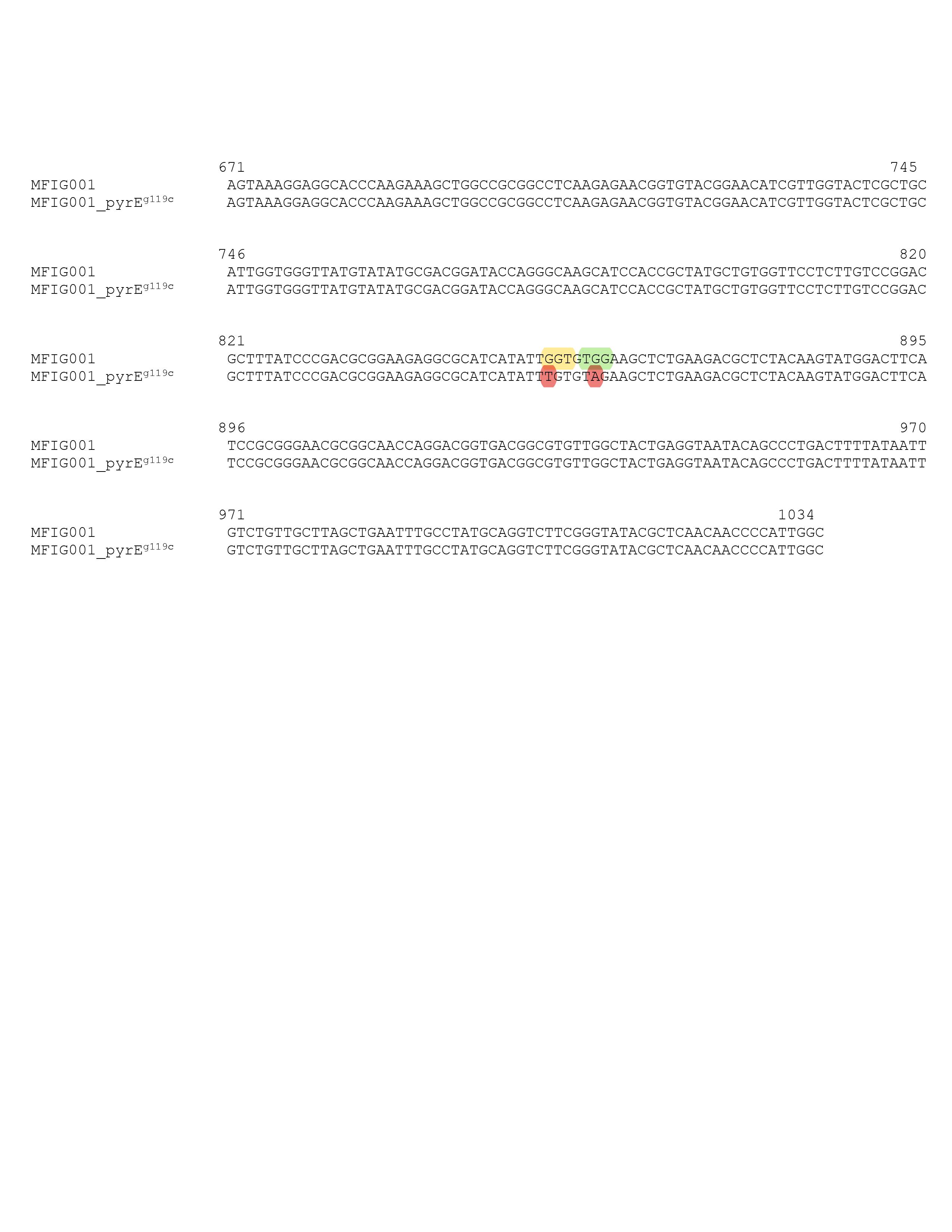

Supplement: Supplemental Material [file TEMI_A_2034485_SM8816.jpg]

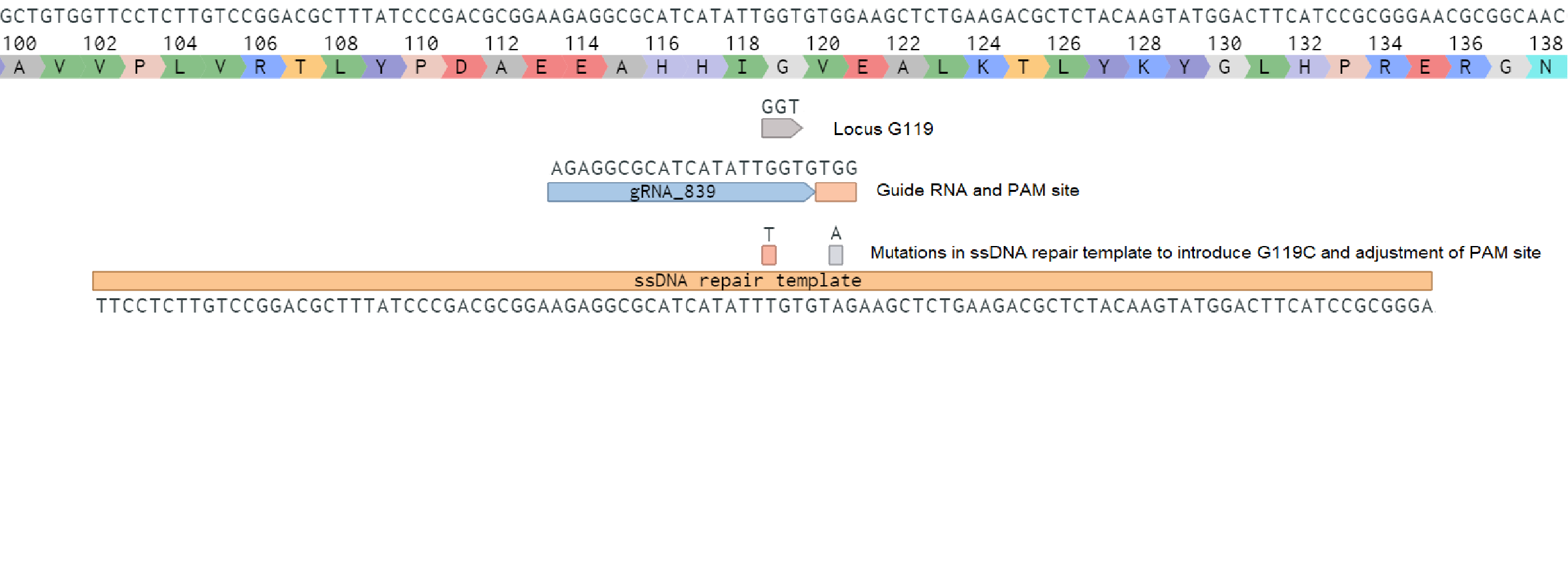

Supplement: Supplemental Material [file TEMI_A_2034485_SM8795.tif]
